# Inferring Signatures of Positive Selection in Whole-Genome Sequencing Data: An Overview of Haplotype-Based Methods

**DOI:** 10.3390/genes13050926

**Published:** 2022-05-22

**Authors:** Paolo Abondio, Elisabetta Cilli, Donata Luiselli

**Affiliations:** 1 Department of Cultural Heritage, University of Bologna, Via Degli Ariani 1, 48121 Ravenna, Italy; elisabetta.cilli@unibo.it (E.C.); donata.luiselli@unibo.it (D.L.); 2 Laboratory of Molecular Anthropology and Center for Genome Biology, Department of Biological, Geological and Environmental Sciences, University of Bologna, Via Selmi 3, 40126 Bologna, Italy; 3 Fano Marine Center, The Inter-Institute Center for Research on Marine Biodiversity, Resources and Biotechnologies (FMC), Viale Adriatico 1/N, 61032 Fano, Italy

**Keywords:** positive selection, whole-genome sequencing, haplotype-based methods, haplotype, linkage, selective sweep, haplotype homozygosity, haplotype composition, haplotype frequency, statistics

## Abstract

Signatures of positive selection in the genome are a characteristic mark of adaptation that can reveal an ongoing, recent, or ancient response to environmental change throughout the evolution of a population. New sources of food, climate conditions, and exposure to pathogens are only some of the possible sources of selective pressure, and the rise of advantageous genetic variants is a crucial determinant of survival and reproduction. In this context, the ability to detect these signatures of selection may pinpoint genetic variants that are responsible for a significant change in gene regulation, gene expression, or protein synthesis, structure, and function. This review focuses on statistical methods that take advantage of linkage disequilibrium and haplotype determination to reveal signatures of positive selection in whole-genome sequencing data, showing that they emerge from different descriptions of the same underlying event. Moreover, considerations are provided around the application of these statistics to different species, their suitability for ancient DNA, and the usefulness of discovering variants under selection for biomedicine and public health in an evolutionary medicine framework.

## 1. Introduction

After the Out of Africa event (around 60–70 kya), modern humans spread across the world, colonizing regions with new environments (locally new food resources, climate, and pathogens) and adapting to cope with the challenges induced by these new selective pressures that have left molecular signatures in the genomes of present-day populations [1,2,3].

The ability to accurately detect and quantify the influence of selection from genomic sequence data enables a wide variety of insights, ranging from understanding historical evolutionary events to characterizing the functional and disease relevance of observed or potential genetic variants [1,2,3]. The genomic footprint of positive selection is generally characterized by long high-frequency haplotypes and low nucleotide diversity in the vicinity of the adaptive locus, and statistical tests for the detection of these signatures have been developed since before the inception of the whole-genome sequencing era, more than 20 years ago [1,2,3]. By assigning statistical scores to single nucleotide variants contextualized in their haplotypic surroundings, these tests allow one to detect ongoing, recent, and even ancient instances of selection on either ancestral or derived alleles, according to the selective sweep model taken into consideration [4,5]. In fact, candidate loci under selection may be significant drivers or contributors to current advantageous but also pathological phenotypes, and selection tests can pinpoint the mutations possibly responsible for changes in the regulation of gene expression, as well as in protein structure and function.

The present article provides an overview of the key concepts that allow to one understand the origin and nature of haplotypes, their application to population genomic studies, and how they carry signatures of selection. Then, three notable viewpoints (pattern of haplotype homozygosity, variation in haplotype composition, and change in haplotype frequency) are introduced to contextualize how different interpretations of the same underlying phenomenon (that is, genetic similarity at the same locus in different individuals belonging to the same population) have been used to develop, over the years, statistical frameworks that are based on the knowledge of haplotypes and linked variants. Finally, after presenting several statistical tests, standalone programs, and packages, closing considerations around the applicability, usefulness, and importance of the information provided by these statistical tests are offered.

## 2. Haplotypes, Population Genomics, and Signatures of Selection

### 2.1. How Do Haplotypes Arise?

Meiosis is a characteristic event that leads to the production of haploid gametes in sexually reproductive diploid organisms, such as humans [6]. A peculiar feature of this process, which is typical of germ cells, is the alignment of homologous chromosomes (one copy of which was inherited from one parent and one from the other parent) along the central axis of the cell before the first round of division, with a subsequent exchange of genetic material between them [7,8]. This process of recombination, also called “crossing over”, is what allows the new generation to carry different genetic combinations, increasing the overall diversity and variability of the population: the offspring will carry different combinations of genes than their parents [8]. Its significance, however, is amplified by two main observations. Firstly, genetic material is inherited in chunks, not as single nucleotides, which implies that each nucleotide will be passed on to the next generation surrounded by a specific cluster of variants on the inherited DNA segment. Secondly, recombination is a largely random event (although specific sites exist, which are more prone to recombination) [9,10,11,12,13], which means its rate can be averaged along the genome to estimate a relatively constant probability at any location. This implies that, given any two nucleotides belonging to the same chromosome, their frequency of recombination approximates the physical distance separating them, with sites that are physically close being less susceptible to recombination and therefore more probably inherited together. Along a chromosome, if the probability pAB of finding any two sites A and B together (thus constituting a haplotype, or a block of linked variants that are inherited together) is higher than the combined probabilities of finding them separately, pApB, then the sites are said to be in linkage disequilibrium (LD) [14,15,16]. So, at the population level, for sufficiently distant sites on the same chromosome, the probability of crossover is high enough to destroy any correlation between them, breaking the continuity of the haplotype, which is generally not inherited as a single block of linked variants anymore [17,18,19,20].

### 2.2. Haplotypes in the Context of Population Genomics

Given this background, let it be assumed that a mutation, represented by an alternative nucleotide (or allele) for a variant, may become heritable by appearing in the coding region of a gene, along the genome of a germ cell in an individual. It is also assumed that the product of this gene (e.g., a protein) may be altered in a way that enhances either the reproductive chances of this individual or its survival in an environment, making it a beneficial allele. The implication is that the offspring of this individual, which inherits the positive mutation, will also have a fitness advantage under the same environmental conditions in terms of survival and possibility to reproduce, so that, in time, the population that the starting individual belongs to will be enriched in subjects carrying the same beneficial allele. So, if a mutation provides an evolutionary advantage, its frequency will increase over generations in the context of the same selective pressure (i.e., any environmental cause that may alter reproductive success) [21].

As presented previously, however, variants along a DNA filament are linked and genetic material is inherited in chunks during homologous recombination. Therefore, not only the mutation that is positively selected will increase its frequency across generations but also the neutral variants surrounding it, which are carried along the same segment in a phenomenon called “hitchhiking” [22,23,24,25]. By taking the genomes of individuals from a population and aligning them, the result of this process can be observed as it generates regions (which can be approximated by haplotypes) of high LD and low genetic variability [26]. As recombination breaks the link between variants over time, it is expected that relatively recent adaptive events will be represented by comparatively extended haplotypes, while older selection events will be observed as smaller haplotypes in the overall population [5].

### 2.3. Haplotypes and Selection Events

**Figure 1:**
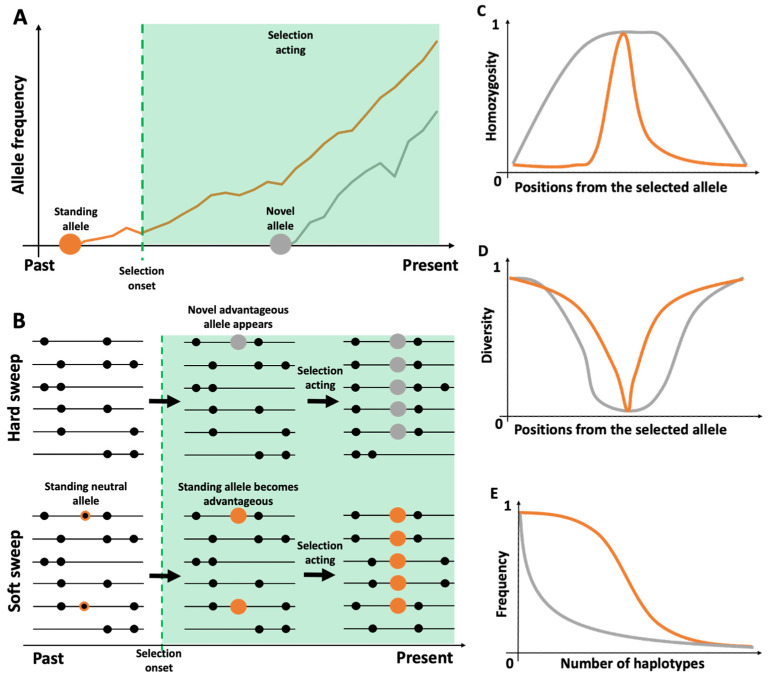
Effect of selection of standing and novel alleles. (**A**) A novel allele, in grey, appears after selection has begun its action and is immediately beneficial, so it will rapidly increase its frequency in the population over time; a standing allele, in orange, already exists in the population as a neutral allele with relatively low frequency, and it increases after becoming beneficial. (**B**) Representation of a hard and a soft sweep, relative to the conditions described for panel A. (**C**) Variation in the pattern of haplotype homozygosity in a region surrounding a central ancestral (orange) or derived (grey) allele under selection. (**D**) Variation in the pattern of haplotype composition in a region surrounding a central ancestral (orange) or derived (grey) allele under selection. (**E**) Decrease in the frequency of each most frequent haplotype, given an increasing number of haplotypes surrounding a standing (orange) or novel (grey) positively selected allele in the population.

As selective pressure is dependent upon the conditions in which a population lives, genetics and environment display a mutual effect, with a change in either one being able to trigger an environment-dependent selective event [2]. As already introduced before, a novel allele (i.e., a genetic change) may arise through mutation, which provides an advantage in the existing environment (Figure 1a, grey). As DNA is inherited in segments, a consequence of the fitness advantage conferred by the new mutation will be an increase of its frequency in the population over generations, together with the surrounding neutral variants on the same genetic chunk (hitchhiking). More specifically, the mutation will possibly appear only once, in a single individual, and therefore will be linked to a very definite neutral background, leading over time to a sharp reduction of genetic variability in a comparatively large haplotype around the mutation (a “hard sweep” model, Figure 1b) [4,5]. Conversely, a prolonged period of change in environmental conditions (e.g., migration to higher altitude or hotter climate; consistent exposure to new pathogens; a permanent dietary alteration) could act as a selective pressure on an already existing polymorphism that was previously neutral (Figure 1a, orange). This now positive allele, which confers an evolutionary advantage in terms of fitness, is already present in several members of the population and therefore is associated with a more varied neutral genetic background. Consequently, several different haplotypes in the population will be surrounding the advantageous mutation, and over time the reduction of genetic variability in that segment will be less marked (“soft sweep” model, Figure 1b), with at most only the variant under selection showing complete loss of variability (i.e., fixation) in the population [4,5].

Most haplotype-based methods can distinguish between hard and soft sweeps, if the ancestral and derived states of the alleles for each variant making up the haplotype are known. This information can be introduced by comparing the sequences of the individuals under study with non-human reference primate sequences and assuming, at a minimum, a model in which the alleles shared between humans and all other primates are inherited from their common ancestor and therefore are treated as ancestral. This in turn implies that the derived allele has appeared along the human lineage at a later time and is therefore more recent. One can then assume that signals of selection associated with a soft sweep will be characteristic of older, ancestral alleles that became beneficial after a change in environment (Figure 1a, orange), while signals associated with a hard sweep will be the signature of more recent, derived alleles that were advantageous and underwent selection immediately after their appearance in the human lineage (Figure 1a, grey).

Of course, given these two extreme models, one must acknowledge the existence of a range of other possible influences which result in intermediate conditions, called “incomplete selective sweeps”. For example, the time at which the selective event has taken place in the past, as well as its intensity and origin, has an impact on the genetic variability surrounding the site under selection and produces intermediate changes in variability along the inherited DNA segment. Moreover, it has been shown that sometimes the two alleles of the same variant are both beneficial when expressed together in heterozygosity, which over a long time generates segments with average variability surrounding a variant with both alleles at about the same frequency in the population (balancing selection). Finally, it is important to highlight that, in contemporary human population genomics, most of the phenotypes are being approached as complex traits, in which several genes contribute to the outcome of intricate metabolic and regulatory processes [4,27,28,29]. However, smaller genetic contributions from a high number of interacting genes (and gene products) prove very difficult to model explicitly, even though exciting results have been obtained in recent years by combining genomic scans for positive selection, using several of the methods presented here, with network-based approaches on metabolic pathways [30,31,32,33].

## 3. Investigating the Effect of Positive Selection in a Genomic Region

The effect of a hard or soft sweep on a genetic region is mirrored by the local change in the variability of that segment across the whole population under study, so that pattern differences in an area surrounding the variant of interest may suggest the possibility of selection on a de novo mutation, rather than selection on an already standing variation. However, the same event (local change in variability) may be analyzed in at least three different ways, which provide the basis for several haplotype-based tests that allow for the scan of entire genomes for signatures of positive selection, as listed in Table 1.

### 3.1. Pattern of Haplotype Homozygosity

Given a genomic region centered around a variant with an allele under selection and taken as a reference haplotype for that region (which can belong to the population or come from a previous study), one can easily compute for each single variant the proportion of the alleles belonging to the reference haplotype that are found in the population. Given the presence of LD, it can be expected that this proportion will be near one (close to fixation) for sites around the variant of interest, then it will reduce the more a site is far from the selected variant [18,52,53]. This implies, in turn, that starting from the central polymorphism and considering increasingly bigger segments, the probability of finding the same allele at the same position in any two haplotypes will decrease and selecting two identical haplotypes at random will become more and more improbable. This decay of haplotype homozygosity in a population is a crucial characteristic of selective events that has been extensively exploited to build tests for positive selection (Figure 1c and Table 1).

Sabeti and colleagues [34] were the first to develop a powerful LD-based method to detect positive selection by taking small groups of rarely recombining single nucleotide polymorphisms (SNPs) in regions of interest to build core haplotypes and then observing the decay of LD at increasingly distant SNPs. This method, called “long range homozygosity” (LRH), detects the transmission of an extended haplotype without recombination (extended haplotype homozygosity, EHH) and implies finding a core haplotype with a combination of high frequency and high EHH, as compared with the other core haplotypes in the same region that serve as internal controls [34]. The authors also affirm that this test can be used to scan the entire human genome for signatures of positive selection, without prior knowledge of a specific variant or selective advantage for a population [34]. The method seems particularly efficient for selective events over the last 10,000 years (around the introduction of domestication and agriculture, with the spread of new infectious diseases, food sources, and cultural/social structures), as these may have left clear signals of long-range LD (0.25 centiMorgans, cM), which should be distinguishable from the shorter extent of LD (0.02 cM) for common haplotypes in the genome [34].

This method for intra-population scanning was then adapted into the whole-genome long-range haplotype test (WGLRH) [35]. Starting from the same premises of the LRH, the WGLRH algorithm identifies core haplotypes of non-recombining SNPs along the genome and computes EHH for an extended segment of 500 kilobases (kb) around them [35]. However, instead of using the other core haplotypes in the same location directly as controls, each haplotype is compared to the genome-wide distribution of relative EHH (rEHH) for haplotypes of similar frequency, where rEHH is the EHH of one core haplotype relative to other core haplotypes in a core region, adjusted for their frequency in the population [35]. This is performed under the assumption that most markers in the human genome are neutral for autosomal chromosomes, and the core haplotypes that experienced recent positive selection should be larger and have higher rEHH when compared to neutral core haplotypes with a similar frequency [35]. So, the genome-wide distribution of rEHH for the core haplotypes with similar frequencies to a core haplotype of interest is used as the distribution of rEHH under neutral conditions [35]. Compared with LRH, the use of genome-wide haplotypes to infer the distribution of LD under neutrality provides a realistic and more computationally efficient solution than modelling neutral distributions from scratch [34,35].

Another method stemming from Sabeti’s LRH was developed for very recent positive selection signals in favor of variants that have not yet reached fixation and is based on the observation that, when selection is acting, the area under the curve obtained by plotting the distance from the core SNP against the decay of EHH is bigger than under neutrality, as in this condition haplotype homozygosity extends much further away from the variant under selection [37]. The integrated haplotype score (iHS) [37] captures this effect by computing the integral of the observed decay of EHH away from a specified core allele in both directions, until EHH reaches a frequency of 0.05. Moreover, it takes into consideration the natural logarithm of the ratio of the integral computed separately around the ancestral and the derived allele, so that, if the EHH decay is similar around the ancestral and derived alleles, iHS will be zero and no selective pressure will be acting [37]. Conversely, positive statistical values will be considered indicative of selective pressure acting on the ancestral allele, while negative values will suggest the influence of selective pressure on the derived allele [37].

A single-population haplotype-based statistic that is somewhat analogous to iHS was introduced to tackle possible incomplete hard and soft selective sweeps [39]. The number of segregating sites by length (nSL) considers all couples of haplotypes carrying the ancestral (or derived) allele for a variant of interest and computes the maximum number of consecutive segregating sites, over which the two haplotypes are identical by state (IBS) [39]. Then, it averages this value over all the pairs of haplotypes carrying the ancestral (or derived) allele tested for the same variant. For each genetic site, nSL is the natural logarithm of the ratio between the average number of segregating sites around the ancestral allele and around the derived allele, and this metric shares the same behavior seen previously for the iHS test [39]. The main difference is that nSL uses segregating sites as a proxy for distance, while the iHS statistic uses the recombination distance directly [37,39]. Comparing this statistic with several other tests for positive selection, the authors verified that nSL is robust to demographic variables such as population growth, bottleneck events, and population structure, as well as to changing recombination and mutation rates (to which the test is blind) [39]. Moreover, its power is extremely elevated (almost 100%) even at very low (0.001–0.1) or very high frequencies of the allele under selection, especially in the case of a hard sweep [39]. Since, in neutral models, low frequency alleles are generally younger in origin and are associated with longer haplotypes than higher frequency alleles, these tests can also be standardized to obtain a distribution with mean zero and variance one regardless of allele frequency at the core SNP [34,35,37,39].

With the advent of dense datasets of genetic variants, tests based on the decay of EHH have also been developed for the comparison of two populations, with the assumption that the same variant may be differentially selected in diverse groups and allows one to discover selected alleles that have swept to near fixation in a population [36]. Computing cross-population EHH (XP-EHH) for two groups A and B at a core allele X and up to an allele Y proximal or distal to the centromere involves integrating the area under the EHH curve with respect to the distance between X and Y, to obtain integrated values IA and IB. The cross-score for said core allele X will be the natural logarithm of the ratio between IA and IB [36]. As with iHS and nSL, the score will be zero if the same decay of homozygosity is observed in both populations; it will be positive if a selective pressure has acted on the allele X preferentially in population A, and it will be negative if selection is stronger in population B [36,37,39]. Similarly, the XP-nSL statistics has been recently introduced for the detection of local adaptation by comparing haplotype patterns between two populations around the same allele of interest, and it has the power to detect both ongoing and recently completed hard and soft sweeps [38].

### 3.2. Change in Haplotype Composition

Given a genomic region centered around a variant with an allele under positive selection, one can assume that the frequency of the selected allele (and of those in high LD with it) will be close to one in the population while, moving increasingly away from the variant of interest, the association between variants will diminish and the frequency of the most frequent allele will reduce, with a principle similar to the decay of homozygosity presented in the previous paragraphs [18,52,53]. This in turn means that the haplotypes in that region will be almost identical in proximity to the selected position, but their allele content will become increasingly different towards the extremities (Figure 1d). This characteristic has been exploited to develop a powerful test, the derived intra-allelic nucleotide diversity (DIND) [40], which is specifically able to detect classical selective sweeps around de novo mutations (i.e., derived alleles). Like nS_L_, DIND requires that haplotypes around a variant of interest be grouped in two clusters, one carrying the ancestral allele and one carrying the derived allele [39,40]. Then, for the whole length of the segment, each pair of haplotypes is compared and pairwise differences at the same positions are counted; differences between all possible pairs are then summed, normalized by the number of haplotype comparisons, and the score obtained for the group of haplotypes carrying the ancestral allele is divided by the score obtained for the derived allele [40]. The statistics will assume a positive value between zero and infinity, with one being the neutral score where haplotypes around the ancestral and derived allele will have the same proportion of differences over the number of performed pairwise comparisons. It also preserves its power of detection of populations with a limited number of individuals (even less than 10); however, it has the crucial limitation of not performing well with very low allele frequencies (less than 0.2), so that only well-established instances of selective pressure around the ancestral allele will be predominantly recognized [40].

Regarding multiple population comparisons, several intriguing tests have been developed that leverage sequence similarity (even to fixation) along haplotypes in a test population and contrast it with sequence similarity in the same region for a reference population under neutral conditions. Kimura and colleagues [41] developed the rMHH (ratio of most frequent haplotype homozygosity) and rHH (ratio of haplotype homozygosity) statistics to reveal fixed loci under selection in a test population without the actual need of systematic haplotype reconstruction along the whole genome by leveraging the homozygous or heterozygous status of each variant at the level of single individuals. When compared with maxFst (the greatest value of Fst in the same genomic region) on simulated and real data, and 90% detection power was assured for both the statistics, maxFst yielded false positives twice more than rMHH (which provided a maximum Type 1 error of 2%) [41].

### 3.3. Change in Haplotype Frequency

Given a genomic region of fixed size centered around a variant with an allele under selection, the sweep model suggests that not only the variant of interest but also the surrounding neutral ones will be driven towards a high frequency in the population, depending on the intensity of the acting selective pressure and the nature of the advantageous mutation. In turn, this implies that, around de novo alleles (hard sweep [4,5]), a single haplotype may be largely represented in the population; conversely, since already-existing alleles that become advantageous through an environmental change are associated with diverse haplotypic surroundings (soft sweep [4,5]), it may be possible that multiple haplotypes carrying the allele under selection will be at a higher frequency in the group of individuals under scrutiny (Figure 1e).

This observation was used by Hanchard and colleagues [42] to develop a sliding window-based test to detect whole haplotype similarity as an indicator of recent positive selection acting on a point mutation. The approach, called haplosimilarity score (HS), is interesting because it does not need information related to ancestral or derived allele conditions, focusing instead on the allele with the minor frequency, and it can be performed on limited regions instead of scanning the whole genome [42]. Supposing an application of this method to each single point mutation in a genetic dataset, the score associated with each variant will be the sum of the squared frequencies of all the haplotypes of fixed dimension detected around said variant in the population, and its value will range between 1/kmax, where kmax is the number of different detected haplotypes, and 1 [42]. The authors also show that the power of the HS test is comparable to LRH [34] in a wide range of minor allele frequencies and remark that the method could be affected by complex demographic histories [42]. Given the focus on the minor frequency allele, HS seems more adequate at detecting ongoing instances of recent, strong positive selection around an allele that is still increasing in frequency in the population [42].

Leveraging the same observations about whole haplotype frequency, Garud and colleagues [44,54] introduced an extended suite of tests that will be addressed here as “H statistics”, which is based on high-frequency haplotypes. In particular, the H1 statistic can be considered a generalization of the HS test seen previously, as it considers the sum of squares along all haplotypes found around all alleles of a selected variant, instead of considering just the minor frequency allele. Values of H1 are expected to be particularly high for hard sweeps, with only one adaptive haplotype at high frequency in the sample [44]. Thus, H1 is an intuitive candidate for a test of neutrality versus hard sweeps, where the test rejects neutrality for high values of H1. This approach gives more weight to recent events of hard sweep, where the contribution of rare, low frequency haplotypes becomes insignificant when compared to that of the single prevalent haplotype, which may well have gone towards fixation in the population [44]. In fact, as sweeps become softer and the number of haplotypes increases, the relative contribution of individual haplotypes towards H1 decreases, and the power of the test is expected to decrease. However, one can also consider a second, related test called H2, which is the same as H1 but excluding the frequency of the most frequent haplotype. By deliberately removing this contribution, in the case of a hard sweep one would obtain a very low value of H2, given that the remaining haplotypes have much lower frequencies in the population; however, this reduction would be less and less noticeable in the case of a soft selective sweep with an increasing number of haplotypes, as the contribution of the most frequent one may be comparable to that of the second most frequent one and, at its most extreme, several haplotypes under selection may provide similar contributions [44,54]. So, considering the ratio between H2 (the sum of the squared frequency of the haplotypes, excluding the most frequent) and H1 (the sum of the squared frequency of all haplotypes) could be a better indication of selection around a novel mutation (hard sweep, H2/H1 close to zero) rather than a pre-existing allele (soft sweep, H2/H1 close to one) [44]. However, this also depends on how many different haplotypes are found, which in turn depends on the size of the segment considered as a haplotype. Garud and colleagues also introduced another notable statistic, H12, which sums the frequencies of the first and second most frequent haplotypes and treats them as if they were one single object [44]. This would have a small effect in the case of a hard sweep, as the small contribution of the second most frequent haplotype should be close to negligible and the value of H12 should be very similar to the value of H1. However, in the case of a soft sweep, the contribution of the first and second most frequent haplotypes may be comparable in size, and their squared sum would be much bigger than the value of H1. This suite of haplotype homozygosity tests delineates an intuitive and easy way to discern between hard and soft selective sweeps without relying on ancestral information; moreover, its performance as compared with iHS suggests that H12 better recognizes recent, strong selective sweeps and is more powerful in identifying soft sweeps [44].

As with the decay of haplotype homozygosity, cross-population tests have been developed as well in recent years (Table 1). Comparative haplotype identity statistics [43] is a haplotype-based method that assesses population-specific instances of local adaptation, considering the possibility that an allele may have undergone selection several times in different populations and that it may be under fixation in a population but still variable in another one. Given a variant X and a threshold length L, the test computes the pairwise comparison of all segments in each population centered around the variant and sums the length of the largest haplotype block for each comparison, if it is bigger than L. Then, haplotype sharing in the population of interest, P1, is divided by the haplotype sharing in the “reference” population, P2. If P2 = 0, one can use L as the denominator of the division. The authors show that, especially in particular cases such as partial soft sweeps, this test outperforms both XP-EHH (based on the decay of haplotype homozygosity) and Fst (a classic measure of population differentiation based on single nucleotide frequency variation), and it can detect ancient selective events as well [43].

Recently, Harris and DeGiorgio [45] developed an alternative version of the H12 statistics that identifies genomic regions under shared positive selection across populations, supposing that the signature of a selective sweep in an ancestral population may remain in its descendants. Given two populations and a variant of interest X, SS-H12 takes into consideration both the overall sharing of haplotypes, centered around X, between them and the different frequencies at which the same haplotype appears in the two populations [45]. By evaluating both the haplotype frequency spectrum and quantifying shared haplotype identity in terms of frequency, SS-H12 properly identifies and differentiates between independent convergent sweeps and true ancestral sweeps, with high power and robustness to numerous demographic variables [45].

### 3.4. Programs and Packages

As presented in the previous subsections, several LD-based and haplotype-based tests for positive selection have been developed over the years, leveraging different aspects of the same underlying genetic phenomena that are supposed to incorporate and describe ancient, recent, and ongoing selective events around either novel or standing variations (Table 1). To highlight the utility and relevance of these methods, it should be noted that, over the last decade, software has been developed for the easy computation of several among the presented tests to facilitate the user in their application (Table 1). For example, the *selscan* self-standing program (https://github.com/szpiech/selscan) [49,55] was introduced to perform EHH-based scans for positive selection: its most recent version currently implements EHH, iHS, XP-EHH, nSL, XP-nSL, and H12. Similarly, the *hapbin* program [51,56] was developed to easily compute iHS, EHH, and XP-EHH. Interestingly, this program obtains the same results as *selscan*, but the computational approach used makes it up to 3.400 times faster, especially when the population under study has a relatively low number of individuals (25 to 100). The *rehh* R package [46,57] and its upgrades for large datasets [47] and for unphased/unpolarized data [48] have also been introduced to perform the EHH-based scans for positive selection iHS, XP-EHH, and Rsb [58] (the latter does not require haplotype reconstruction, so it is not described in the context of the present work). By measuring the false discovery rate in simulated whole-genome scans and quantifying the overlap of inferred candidate regions in empirical data, the authors find that phasing information is necessary for accurate estimation of within-population statistics (except in the case of very large samples) and of cross-population statistics for small samples, while ancestry information is of lesser importance in both cases [48]. Recently, a novel open-source program, *lassip* [50,59], was published with a focus on scans for positive selection based on a haplotype frequency spectrum. The standalone program implements various haplotype frequency spectrum statistics useful for detecting hard and soft selective sweeps in genomes, including SS-H12 [45], the H statistics [44], as well as the genotype-based unphased versions of the latter (called G statistics) [60]. The authors show that implementing a likelihood-based approach based on explicit demographic models for population evolutionary history improves the discovery of both hard and soft selective sweeps in haplotype-based data, as does accounting for distortions in the spatial distribution of the haplotype frequency spectrum along the genome, relative to genome-wide expectation taken as neutrality [50].

## 4. Considerations around Haplotype-Based Tests for Positive Selection

### 4.1. Appropriateness for Different Types of Genetic Variants

As presented in the previous sections, haplotype-based tests for positive selection are usually applied to biallelic SNVs or single-base mutations with only two alleles. Nonetheless, it is known that several types of variation along the genome have been under selection: microsatellites or short tandem repeats [61,62,63], copy number variants (CNVs) [64,65], sub-microscopic structural variants (SVs) [66,67], and transposable element (TE) insertions [68,69,70] all show definite signatures of population differentiation that point towards positive selection events. However, the application of LD-based methods built on haplotype reconstruction requires that non-SNP objects are managed with extreme care, because of their multi-allelic nature: a genomic scan for positive selection must be able to recognize and distinguish among essentially different genetic elements with disparate lengths. One possible solution could be to consider the LD between structural variants and nearby point mutations and take advantage of the associated single nucleotide variant as a proxy for the structural variation [65,71]. Indeed, there is almost no literature exploring the effectiveness of such methods on variants that are not SNPs [72,73] or the development of specific algorithms for the detection of signatures of selection around them. 

### 4.2. Applicability across the Tree of Life

It is important to remember that *Homo sapiens* has never been the only living species characterized by repeated events of migration, colonization, and expansion throughout its existence: all present forms of life survive because they have evolved by adapting to changing habitats, new climate conditions, and different diets, while settling in radically diverse environments. Accordingly, characteristic signatures of positive selection could be hypothetically retrieved in all extant species and populations, with haplotype-based scans. Indeed, several of the methods presented in this manuscript have been applied not only to humans but to many other living organisms and for different purposes. Economically relevant species of animals and plants, such as pigs (*Sus domesticus*) [74], cattle (*Bos taurus*) [75,76,77], yaks (*Bos grunniens*) [78], sheep (*Ovis aries*) [79], horses (*Equus caballus*) [80,81], and tomatoes (*Solanum lycopersicum*) [82] have been researched mainly for commercially favorable instances of selection; pathogens and disease vectors, including *Plasmodium falciparum* [83,84] and mosquitoes (*Anopheles gambiae*) [85], for their rapid evolution and resistance to toxic compounds; companion animals, such as dogs (*Canis lupus familiaris*) [86,87], were the focus of studies to understand the influence of domestication in close contact with humans. For example, Zorc and colleagues [74] applied the iHS test on both SNPs and microsatellites to reveal that six autochthonous Balkan pig breeds present different genes under positive selection, with particular reference to reproductive traits (number of offspring, sperm quality, early pregnancy), muscle mass, fat metabolism, and disease resistance. Seo and colleagues [75] applied iHS and EHH on an unselected Korean cattle breed and compared the results with the signatures given by a population (KPN) that underwent a 30-year-long artificial selection program for breeding traits, including total weight and back fat thickness. Significant signatures of selection were detected for KPN variants in 44 genes, with significant association of variants in chromosome 14 with the aforementioned breeding traits, while metabolic pathways related to selective signatures on chromosome 13 mainly impact energy metabolism and feed efficiency. They also verified that the allele under selection for KPN was derived in most instances [75]. The study performed by Zhao and colleagues on 163 tomato plants from three groups also used the iHS statistic to reveal 24 positive selective sweeps associated with tomato quality traits, including an improvement of tomato fruit weight and sugar metabolism [82]. Using *P. falciparum* isolates from young subjects in the Plateaux Region of Togo, Kassegne and colleagues highlighted that 10 red blood cell invasion-related antigen genes show signatures of positive selection, together with 134 immune-related and adhesion genes and eight genes positively selected for drug resistance [83]. Lucas and colleagues took advantage of data from the “*Anopheles gambiae* 1000 Genomes Consortium” and applied EHH to identify 44 CNVs subjected to positive selection: the genes found were enriched for families involved in metabolic insecticide resistance [85]. Finally, Schlamp and colleagues carried out an interesting comparative analysis of different statistical methods (including iHS, nSL, and the H statistics) for the detection of signatures of positive selection in 25 dog breeds [86]. Testing for 12 known loci (positive controls) that are likely causal of breed-specific traits (body size; coat color; hair, lip, ear, and snout shape and length), their work revealed that not all tests are able to detect the same signatures of positive selection and, on the other hand, that some genes are under selection in some breeds but not in others [86].

### 4.3. Pertinence to Ancient DNA

Hypothetically, haplotype-based statistics for detecting instances of positive selection may be applied not only on samples of living organisms but also on DNA collected from ancient remains of extant and extinct populations. However, ancient DNA comes with its own set of problems [88,89,90,91]: an organism’s DNA degrades over time, it is highly fragmented, often modified chemically, and it is usually retrieved in low quantities, even with the best extraction protocols. It is much more challenging to phase ancient DNA because endogenous reads are rare and short. After reassembling the reads, some regions of the genome may only have a couple or less reads mapped to it. Although variants may be found within two reads, it is difficult to distinguish real genetic variants from false variants produced by deamination, especially when the genomic libraries are not repaired with uracil DNA glycosylase (UDG) treatment and/or hybridization capture methods are not applied [92]. Due to its low information, the underlying haplotypes of ancient DNA cannot be discerned (unphased) [93,94]. As these algorithms require knowledge (or at least a hypothesis) of LD and haplotype reconstruction, variant density along the genome is crucial for the performance of several tests, and for low quality samples it may prove very difficult to properly perform them, ultimately impairing their application on ancient samples. Single nucleotide, genotype-based tests for differentiation usually provide much more informative insights on possible existing selective pressures acting on ancient populations. Moreover, the number of considered individuals in a population is equally important, as the sample may not be a real representative of the variability existing in a group and this also has repercussions on the performance of several population-oriented haplotype-based tests for selection.

### 4.4. Relevance to Human Medicine and Public Health

As introduced in the previous sections, *Homo sapiens* experienced consistent migration events over tens of hundreds of years, with smaller populations periodically separating from the main group and colonizing new territories and consequently being exposed to new environments [1,2,3]. Both previously existing and novel alleles underwent multiple instances of positive selection in different populations, over relatively long periods of time. Indeed, different combinations of the methods presented here may reveal different instances of positive selection: from hard to soft selective sweeps and from ongoing to recent to ancient onset. This also implies that individuals of different ethnicity and ancestrality, living different lifestyles in different environments, may have developed distinct adaptations that make them more or less able to metabolize particular substances, such as specific foods or medical compounds. Many modern human diseases exist because populations have not adapted to changing environments or previous adaptations led to trade-offs between health and fitness (evolutionary medicine approach) [95,96,97,98]. However, disease is not just a product of the modern world. As long as there is phenotypic variation, disease is inevitable; some individuals will be better suited to some environments (and thus healthier) than others [94]. Moreover, it is argued that the recent rapid changes introduced with industrialization and globalization may have affected the contemporary generations, so that traits that have been adaptive in specific environments may have become dis-adaptive and at the basis of what are considered “lifestyle diseases” and “diseases of affluence” [95,98]. In this context, haplotype-based methods may reveal loci under selection associated with pathological phenotypes in cohorts of individuals affected by specific diseases, revealing the importance of evolutionary genomic methodologies in the biomedical field [66,67,95,96,97,98].

## Figures and Tables

**Table 1:** List of popular methods, algorithms, statistics, and packages for detecting haplotype-based signatures of positive selection in population-wide sequencing data. EEH: extended haplotype homozygosity; LRH: long-range haplotype; iHS: integrated haplotype score; nSL: number of segregating sites by length; XP-EHH: cross-population extended haplotype homozygosity; XP-nSL: cross-population number of segregating sites by length; DIND: derived intra-allelic haplotype diversity; rMHH: ratio of most frequent haplotype homozygosity; HS: haplosimilarity score; CHI: comparative haplotype identity; SS-H12: H statistic for shared selection; REHH: R package for extended haplotype homozygosity-based test computation.

	Within Population	Between Populations
Haplotype homozygosity	EEH (LRH) [34]	
	WGLRH [35]	XP-EHH [36]
	iHS [37]	XP-nSL [38]
	nSL [39]	
Haplotype diversity	DIND [40]	rMHH [41]
Haplotype frequency	HS [42]	CHI [43]
	H statistics [44]	SS-H12 [45]
Programs and packages	rehh [46,47,48]	
	selscan [49]	
	lassip [50]	
	hapbin [51]	

## Data Availability

Not applicable.
